# Challenges of ECMO use for severe trauma: a narrative review

**DOI:** 10.1186/s41065-025-00611-4

**Published:** 2025-11-22

**Authors:** Xin-Liang Zhang, Ying-Qi Zhang, Zhi-Yong Hou, Wei Chen

**Affiliations:** 1 Emergency Department, The Third Hospital of Hebei Medical University, Shijiazhuang, Hebei Province 050051 China; 2 Trauma Emergency Center, The Third Hospital of Hebei Medical University, No. 139, Ziqiang Road, Qiaoxi District, Shijiazhuang, Hebei Province 050051 China

**Keywords:** Complication management, Extracorporeal membrane oxygenation, Individualized treatment, Prognostic factors, Severe trauma

## Abstract

**Background:**

Patients with severe trauma are at high risk of developing life-threatening complications, including acute respiratory failure and circulatory collapse. Extracorporeal membrane oxygenation (ECMO) offers critical support when conventional therapies fail. This narrative review aimed to analyze the clinical application of ECMO in patients with severe trauma, focusing on indications, complications, prognostic factors, and future directions.

**Methods:**

A comprehensive literature search was conducted to identify studies published between January 2000 and April 2025. Sources included original research articles, systematic reviews, and meta-analyses, supplemented by relevant clinical guidelines and expert consensus statements pertaining to ECMO use in trauma care.

**Results:**

Veno-venous ECMO has been primarily utilized in patients with trauma-associated acute respiratory distress syndrome, while veno-arterial ECMO has been employed in the management of cardiogenic shock and cardiac arrest. Reported survival rates were 72.3% for veno-venous ECMO and 39.0% for veno-arterial ECMO. ECMO has also been used in patients with traumatic brain injury and those undergoing extracorporeal cardiopulmonary resuscitation, although randomized controlled trial data remain limited Major complications include infection, hemorrhage, and thrombosis, which require coordinated prevention and management strategies. Prognostic tools such as the New Injury Severity Score, Simplified Acute Physiology Score III, and Sequential Organ Failure Assessment score are used in clinical evaluation, though trauma-specific models are still lacking.

**Conclusion:**

ECMO offers a supportive treatment modality in the management of patients with severe trauma. To improve clinical outcomes, further development of trauma-specific decision tools, multicenter studies, and standardized protocols for anticoagulation and infection control is necessary to support individualized care.

## Background

According to the Global Burden of Disease Study 2021, major trauma accounts for approximately 5 million deaths annually worldwide, representing a leading cause of mortality and long-term disability [1]. It significantly impacts quality of life and places substantial strain on healthcare systems [2, 3]. Epidemiological data show that early mortality in severe trauma ranges from 30% to 40%, with craniocerebral injury and hemorrhagic shock identified as the primary contributors. In contrast, late-stage mortality is more commonly associated with acute respiratory distress syndrome (ARDS) and multiple organ dysfunction [4–6]. The condition disproportionately affects young adult males, and its age distribution has remained consistent over the past three decades, underscoring persistent global challenges in trauma care [2, 7].

Extracorporeal membrane oxygenation (ECMO) was initially developed for managing acute respiratory failure [8]. Technological advancements have led to the miniaturization and modularization of ECMO systems, accompanied by improvements in safety and ease of deployment [9]. The 2009 Conventional Ventilatory Support vs. Extracorporeal Membrane Oxygenation for Severe Adult Respiratory Failure trial demonstrated that veno-venous ECMO (VV ECMO) significantly improved outcomes in patients with severe ARDS, achieving a rate of 63% compared to 31% with conventional therapy [10]. A 2023 systematic review and meta-analysis reported an overall survival rate of 65.9% for patients with severe trauma receiving ECMO, with 72.3% survival for VV ECMO and 39.0% for veno-arterial ECMO (VA ECMO), particularly demonstrating benefit in trauma-related ARDS and cardiogenic shock [11].

Patients with trauma present unique challenges due to a high risk of bleeding and coagulopathy, resulting in elevated complication rates compared to general ECMO populations—bleeding (30%–54%) and infection (25%–40%) being the most common [11]. Anticoagulation management remains a major obstacle. However, much of the current evidence originates from retrospective cohort studies, with considerable variability in outcomes and limited external validity [12]. Heterogeneity in the extent of organ dysfunction and the timing of early complications complicates the assessment of ECMO candidacy and optimal initiation timing [13]. Evidence is especially lacking in resource-limited settings and for long-term outcomes (≥ 1 year), such as neurological recovery and multi-organ sequelae in patients with polytrauma. Moreover, standardized protocols for heparin-free ECMO, alternative anticoagulants, and dose titration in patients with coagulopathy are not well established.

This review aims to support clinical decision-making regarding ECMO use in severe trauma by synthesizing current evidence, defining trauma-specific indications, identifying key management considerations, and evaluating prognostic indicators. Additionally, it outlines research priorities, including the development of validated decision-making models, the implementation of high-quality prospective studies, and the standardization of protocols.

## Methods

We conducted a literature search covering the period from January 1, 2000, to April 30, 2025, using the PubMed and Web of Science databases. Search terms included “extracorporeal membrane oxygenation,” “ECMO,” “trauma,” “severe injury,” and “ARDS,” among others. Eligible sources included original research articles, systematic reviews, and meta-analyses. Single case reports and non-systematic reviews were excluded.

Study selection was independently performed by two reviewers. Studies addressing the use of ECMO in severe trauma were included, with a focus on complication management and prognostic assessment.

## Evaluation of ECMO initiation indications in patients with severe trauma

Establishing clear indications for ECMO initiation in patients with severe trauma remains a clinical challenge. These patients often present with complex pathophysiological conditions, including high bleeding risk, multi-organ injury, and rapidly evolving physiological instability. Multi-center retrospective analyses have reported that VV ECMO accounts for 71.4% of cases, while VA ECMO is used in 24.5%, with the remainder involving mode conversion or alternative ECMO configurations [11].

### Respiratory failure (ARDS)

VV ECMO is primarily indicated for patients with trauma-related ARDS, particularly when conventional ventilation strategies such as prone positioning and lung-protective ventilation fail to maintain adequate gas exchange [14]. A 2025 meta-analysis encompassing four studies and 1,526 patients demonstrated a significant reduction in mortality associated with ECMO in trauma-related ARDS (odds ratio [OR] 0.29; 95% confidence interval [CI] 0.14–0.62; *p* = 0.001). The VV ECMO subgroup showed an even greater effect (OR 0.19; 95% CI 0.07–0.53; *p* = 0.002) [15]. However, ECMO was also associated with prolonged intensive care unit (ICU) stays, and no significant differences in complication rates were observed. These findings support the use of ECMO as a rescue strategy, particularly when initiated early in the disease course. However, the current evidence is primarily derived from observational studies (Level B) and has not yet been validated through prospective randomized controlled trials (RCTs), limiting the generalizability of conclusions.

Recommended criteria for initiating VV ECMO include the following: PaO_2_/FiO_2_ < 100, refractory hypercapnia with arterial pH < 7.2, and the presence of a high risk of ventilator-induced lung injury (VILI) [16, 17].

The proposed mechanisms for improved outcomes include: (1) provision of effective oxygenation support; (2) reduction in alveolar overdistension and oxygen toxicity, to VILI; and (3) attenuation of the pulmonary inflammation with promotion of alveolar repair [18, 19].

### Circulatory failure

VA ECMO is primarily used in patients with trauma-related circulatory failure, including traumatic cardiac arrest (TCA), cardiogenic shock, and hemodynamic instability unresponsive to conventional resuscitation efforts [20]. A systematic review reported a survival rate of 39.0% for VA ECMO in trauma, significantly lower than the 72.3% observed with VV ECMO [11].

A 2025 analysis from the TraumaRegister DGU^®^ (*n* = 153) further supported the role of VA ECMO in trauma-related circulatory failure. Indications in this cohort included TCA (38%), cardiogenic shock (33%), and severe thoracic trauma with hemodynamic compromise (25%) [21]. Reported survival rates ranged from 39.0% to 53.0%. Early initiation, defined as within six hours of circulatory collapse, was associated with improved outcomes (OR 0.62; 95% CI 0.41–0.94; *p* = 0.025).

Common criteria for VA ECMO initiation include hypoxemia with a PaO₂/FiO₂ ratio below 100, refractory hypotension with a systolic blood pressure below 90 mmHg, and a cardiac index under 2.2 L/min/m². Independent predictors of mortality identified in multivariate analyses include age greater than 65 years, male sex, severe thoracic trauma with an Abbreviated Injury Scale score of 3 or higher, massive transfusion exceeding 10 units of packed red blood cells, and baseline coagulation dysfunction [21].

Complications remain a major concern in patients with trauma receiving VA ECMO, with reported bleeding rates ranging from 30% to 50%, particularly due to intracranial hemorrhage, and thromboembolic events occurring in approximately 10% to 20% of cases [18]. The TraumaRegister DGU^®^ analysis highlighted the relatively high bleeding risk associated with VA ECMO, estimated at 35% to 50%, and recommended individualized anticoagulation strategies, such as the use of low-dose heparin with a target activated clotting time (ACT) of 160 to 200 s, to mitigate this risk [21]. The proposed mechanisms through which VA ECMO may improve outcomes in trauma-related circulatory failure include restoration of systemic perfusion, stabilization of hemodynamic parameters, and reduction of myocardial workload [18, 20]. However, the current evidence is largely limited to retrospective cohort studies, and additional interventions such as open-chest cardiopulmonary resuscitation have been associated with poorer outcomes [20, 21]. Further prospective studies are required to optimize patient selection, refine anticoagulation protocols, and establish standardized approaches for VA ECMO implementation in trauma care.

### Traumatic brain injury (TBI)

TBI has historically been regarded as a relative contraindication for ECMO due to concerns that systemic anticoagulation could exacerbate intracranial hemorrhage. However, reported survival rates among patients with TBI receiving ECMO range from 54% to 66.1%, which are comparable to outcomes observed in patients without TBI (Level B evidence) [11, 14]. These findings suggest that TBI should not be considered an absolute contraindication to ECMO. Rather, clinical decision-making should be individualized and based on comprehensive assessment, including computed tomography (CT) imaging, stratification of bleeding risk, and continuous neurological monitoring.

### Contraindications and risk assessment

Although the use of ECMO in patients with severe trauma is becoming more common, its initiation continues to require comprehensive, individualized evaluation. Absolute contraindications include uncontrolled active bleeding, particularly in the context of intractable intracranial hemorrhage or elevated intracranial pressure, end-stage malignant disease, irreversible cardiopulmonary injury, and the documented refusal of treatment [22, 23].

Relative contraindications encompass conditions such as severe multiple organ dysfunction syndrome (MODS) with a Sequential Organ Failure Assessment (SOFA) score greater than 12, prolonged mechanical ventilation lasting more than seven days, advanced frailty in older adults, and ongoing coagulation disorders [22–26]. In such cases, risk stratification should be dynamic and incorporate both the selection of the appropriate ECMO modality and a careful evaluation of the potential benefits versus risks of intervention.

### Contraindications and risk stratification for ECMO in severe trauma

While the use of ECMO in patients with severe trauma is increasing, its initiation requires careful, individualized assessment to balance potential benefits against significant risks. According to the guidelines of the Extracorporeal Life Support Organization (ELSO), contraindications to ECMO are classified as absolute or relative, depending on whether the anticipated risks outweigh the expected benefits [27]. Absolute contraindications refer to conditions in which ECMO is unlikely to provide meaningful benefit, whereas relative contraindications require dynamic, multimodal evaluation incorporating imaging, laboratory parameters, and clinical judgment. In patients with trauma, ELSO emphasizes that contraindication assessment should account for trauma-specific factors, such as bleeding risk and the heterogeneity of multiple organ injuries. An analysis of the ELSO registry reported a survival rate of 61% among patients with trauma receiving ECMO, supporting its use following careful candidate selection [27].

Absolute contraindications encompass irreversible or high-risk clinical scenarios in which ECMO is unlikely to be effective. These include uncontrolled active bleeding—particularly intractable intracranial hemorrhage, intracranial pressure greater than 20 mmHg, or abdominal or thoracic hemorrhage not amenable to surgical control. ELSO guidelines specify that major active bleeding, such as ongoing blood loss exceeding 1 L per hour, constitutes an absolute contraindication due to the elevated risk associated with systemic anticoagulation [27]. Additional absolute contraindications include end-stage malignant disease in patients with an estimated life expectancy of less than three months, for whom ECMO may be considered only as a bridge to transplantation but is associated with poor outcomes (survival rate < 20%); irreversible cardiopulmonary injury, such as extensive pulmonary fibrosis or myocardial necrosis without recovery potential; and documented refusal of treatment, including do-not-resuscitate (DNR) orders. These contraindications are based on ELSO registry data and systematic reviews. In trauma populations, bleeding-related contraindications represent the most commonly observed exclusion criteria, accounting for approximately 40% of excluded cases.

Relative contraindications must be determined through individualized risk–benefit analysis. ELSO recommends a multi-parameter framework for evaluation, incorporating imaging studies, such as CT or magnetic resonance imaging (MRI) to assess bleeding risk, laboratory indicators including coagulation function and lactate levels, and clinical scoring systems.

High bleeding risk is one of the most common relative contraindications and may be present within 48 h of recent surgery, in the setting of active gastrointestinal ulceration, or following prior thromboembolic events. In patients with trauma, TBI has traditionally been considered a relative contraindication; however, recent data report survival rates between 54% and 66%, which are comparable to those observed in non-TBI cohorts [11, 27]. For these patients, ELSO recommends stratifying bleeding risk through neuroimaging and employing low-dose anticoagulation protocols, with a target ACT of 160 to 200 s.

Severe multiple organ dysfunction also constitutes a relative contraindication. A high SOFA score is associated with increased mortality; however, it does not preclude ECMO initiation if organ dysfunction is potentially reversible. ELSO guidelines note that a SOFA score greater than 12 requires dynamic monitoring, and that ECMO may be considered in cases with a score below this threshold and evidence of reversible pathology. A meta-analysis reported that patients with trauma requiring ECMO and a SOFA score above 12 had a higher risk of mortality (hazard ratio [HR] 1.8), although early intervention may improve outcomes.

Prolonged mechanical ventilation, particularly for more than 7 days, is another relative contraindication, as it increases the risk of ventilator-induced lung injury and infection. However, ELSO guidelines note that many centers do not consider the duration of ventilation an absolute contraindication. Instead, clinical decisions should be based on factors such as lung compliance and the risk of ventilator-induced lung injury. Evidence suggests that trauma-related ARDS patients receiving ECMO after more than 10 days of ventilation may still achieve a survival rate of 50%.

Advanced age accompanied by severe frailty—typically defined as age over 75 years or a frailty index above 5—is associated with poor prognosis, with survival rates below 40%. In such cases, ELSO recommends a comprehensive comorbidity assessment to support decision-making.

Finally, active coagulation disorders, including disseminated intravascular coagulation or thrombocytopenia with platelet counts below 50 × 10⁹/L, also represent relative contraindications. These conditions require appropriate pre-treatment, such as platelet transfusion, prior to ECMO initiation.

In patients with trauma, risk assessment should incorporate the ELSO trauma consensus guidelines [28]. This includes stratifying injury severity using the New Injury Severity Score (NISS) or Injury Severity Score (ISS), applying SOFA score for dynamic monitoring of multiple organ systems, and engaging in multidisciplinary team (MDT) discussions to support individualized decision-making. For patients with traumatic brain injury and elevated bleeding risk, continuous intracranial pressure (ICP) monitoring combined with a heparin-free ECMO strategy is recommended [27].

A systematic review has highlighted that overlooking relative contraindications may lead to an increased incidence of complications, with bleeding rates rising by as much as 20%. In contrast, careful pre-ECMO assessment has been shown to improve survival outcomes [27]. To minimize procedural risks, ELSO further recommends a 24–48 h period of optimization prior to ECMO initiation, incorporating strategies such as prone positioning and lung-protective ventilation where appropriate [27].

### ECMO initiation timing and prognostic impact

The timing of ECMO initiation has a critical influence on prognosis in patients with trauma, particularly in those with ARDS and circulatory failure. Emerging evidence indicates that early initiation, defined as within six hours of injury, is associated with higher survival rates and fewer complications. In contrast, delayed initiation beyond 24 h has been linked to worse outcomes, often due to progression to MODS or persistent hypoxemia [27].

A 2023 meta-analysis involving 36 studies and 1,822 patients with trauma reported that VV ECMO initiated within six hours of injury was associated with a survival rate of 75.2%, significantly higher than the 56.8% observed when initiation was delayed beyond 24 h. The HR for mortality in the early initiation group was 0.55 (95% CI 0.39–0.78; *p* = 0.001), with the observed benefits attributed to rapid correction of hypoxemia and acidosis, reduced risk of VILI, and prevention of MODS progression [27].

In cases of TCA, the 2025 TraumaRegister DGU^®^ analysis of 153 patients found that initiation of VA ECMO within six hours resulted in a survival rate of 53.0%, compared to 39.0% when initiation occurred later. Early intervention was associated with improved outcomes (OR 0.62; 95% CI 0.41–0.94; *p* = 0.025) [21]. ELSO guidelines recommend an initial period of optimization—typically 24 to 48 h—using supportive measures such as prone positioning and lung-protective ventilation. However, if severe hypoxemia (PaO₂/FiO₂ < 80) or acidosis (pH < 7.2) persists for more than six hours, ECMO initiation should not be delayed [27].

Delayed ECMO initiation, particularly beyond 24 h, is associated with a poorer prognosis. A 2024 multicenter study of 412 patients with trauma-related ARDS found that when ECMO was initiated after more than seven days of mechanical ventilation, the survival rate decreased to 50.2%. Additionally, the incidence of complications such as ventilator-associated pneumonia significantly increased (OR 1.9; 95% CI 1.2–3.0). The underlying mechanisms include ongoing VILI leading to pulmonary fibrosis, and progression to MODS, particularly in patients with SOFA scores greater than 12 (HR 1.8). In patients with TCA or cardiogenic shock, delayed VA ECMO initiation has been associated with myocardial exhaustion and irreversible end-organ damage, with survival rates declining to approximately 30% [21]. Despite these findings, ELSO guidelines clarify that ventilation exceeding 10 days is not considered an absolute contraindication, and ECMO may still be viable in patients with preserved lung compliance and potential for recovery [27].

The prognostic effect of ECMO timing is influenced by trauma severity, baseline physiological status, and ECMO modality. A 2025 single-center study involving 24 patients demonstrated that those receiving ECMO within six hours had a survival rate of 80%, without the need for additional interventions such as open-chest cardiopulmonary resuscitation, compared to a 60% survival rate among those receiving delayed ECMO initiation [28].

Several prognostic indicators have been proposed to support initiation timing decisions. A NISS above 40 has been associated with an increased risk of mortality (HR 2.1; 95% CI 1.4–3.2), while a SOFA score below 12 has been linked to improved outcomes (OR 0.45; 95% CI 0.28–0.72) [21]. Elevated lactate levels, particularly those exceeding 10 mmol/L, have been shown to predict poor prognosis, with an area under the curve (AUC) of 0.92. In this context, ELSO recommends the use of MDT assessment that incorporates NISS, SOFA, and dynamic imaging such as CT, particularly for monitoring intracranial pressure in patients with TBI (target ICP < 20 mmHg) [23, 27].

Clinically, early ECMO initiation within six hours has been associated with substantial improvements in survival among patients with trauma-related ARDS and TCA, with reported survival rates of 75.2% for VV ECMO and 53.0% for VA ECMO [11, 21]. Nonetheless, the resource demands and risk of complications, particularly bleeding (30%–50%), must be carefully considered. While delayed initiation may be appropriate in selected cases, vigilance is required to monitor for signs of MODS progression and ventilator-associated complications. Future research should prioritize the definition of optimal initiation windows—potentially exploring ultra-early ECMO initiation within four hours—and the development of trauma-specific prognostic models to enhance patient selection and clinical outcomes.

## Management of ECMO complications and innovative strategies

In patients receiving ECMO support following trauma, the most commonly encountered complications include infection, hemorrhage, and thrombosis. These adverse events have a direct impact on treatment safety and are closely associated with survival outcomes [29]. Effective management requires the implementation of both standardized and individualized strategies, with particular emphasis on precise anticoagulation protocols and tiered approaches to prevention and control. Optimizing these strategies is essential for improving prognosis in this high-risk population.

### Infection: high incidence rate, requiring systematic prevention and control

The incidence of ECMO-associated infections ranges from 25% to 50%, with ventilator-associated pneumonia and bloodstream infections being the most commonly reported complications [30]. These infections are associated with prolonged ECMO support duration, extended ICU stays, and increased mortality, with a reported HR of approximately 1.8. Patients with trauma are particularly susceptible to infection compared to the general ECMO population, due to extensive tissue injury, multiple organ dysfunction, and trauma-induced immunosuppression [29].

Systematic prevention and control strategies are essential to mitigate infection-related complications. Routine microbiological monitoring is recommended, including blood cultures every five to seven days, accompanied by serial measurements of inflammatory markers such as C-reactive protein and procalcitonin. During the early critical phase, empirical antimicrobial therapy should be initiated with a broad-spectrum β-lactam antibiotic combined with coverage for gram-positive organisms. Antimicrobial regimens should be adjusted based on susceptibility testing results within 48 to 72 h.

Emerging evidence suggests that microecological interventions may offer additional benefits. The administration of probiotics, particularly *Lactobacillus* species, has been associated with a reduced incidence of enteric infections in critically ill patients undergoing ECMO [30, 31].

### Hemorrhage and thrombosis: management requires a dynamic balance

Hemorrhage is the most commonly reported complication in patients with trauma receiving ECMO, with reported incidence rates ranging from 35% to 54.2% [21, 28]. Common sites of bleeding include cannulation sites, surgical wounds, and intracranial regions. The risk is exacerbated by trauma-induced coagulopathies such as disseminated intravascular coagulation and thrombocytopenia, which, when combined with the anticoagulation requirements of ECMO, significantly increase the likelihood of bleeding. This is particularly relevant in patients with TBI [11, 21].

A 2025 single-center study involving 24 patients with trauma reported a blood transfusion requirement in 54.2% of cases. Notably, no significant increase in intracranial hemorrhage was observed in the subgroup of patients with TBI (*n* = 7), supporting the feasibility of using low-dose anticoagulation strategies, such as heparin with an ACT target of 160–200 s [28]. Similarly, data from the TraumaRegister DGU^®^ (*n* = 153) indicated a bleeding rate of 35%–50% among patients receiving VA ECMO, with intracranial hemorrhage accounting for 10%–15% of cases [21]. To reduce the risk of severe bleeding events, intensified monitoring of coagulation parameters, including ACT, activated partial thromboplastin time, and platelet counts, is recommended, along with the use of individualized anticoagulation protocols such as heparin-free ECMO or alternative agents like bivalirudin.

Thrombotic complications are also a significant concern, with deep vein thrombosis (DVT), pulmonary embolism, and oxygenator thrombosis occurring in approximately 12.5% to 18% of trauma ECMO cases [21, 28, 29]. The underlying risk is amplified in patients with trauma due to hypercoagulability and vascular endothelial injury, factors that distinguish them from the general ECMO population [29]. Single-center data have documented a DVT incidence of 12.5% and limb ischemia in 8.3% of cases [28]. A meta-analysis further reported a hazard ratio of 1.6 (95% CI, 1.2–2.1) for thrombotic events among trauma ECMO recipients [29]. The risk of thrombosis is higher with VA ECMO than with VV ECMO, likely due to the distinct hemodynamic characteristics of the former, with an odds ratio of 1.4 [21].

To reduce thrombotic risk, recommended strategies include routine ECMO circuit inspections, screening for DVT using vascular ultrasound, and dynamic adjustment of anticoagulation protocols. Targeting an ACT of 160 to 200 s or using low-dose heparin may be beneficial in high-risk patients. However, in patients with TBI, no specific anticoagulation strategy has been shown to reduce the incidence of thrombosis, and no cases of DVT or DIC were reported in this subgroup [28]. These findings underscore the importance of achieving a careful balance between thrombosis prevention and bleeding risk in the management of patients with trauma undergoing ECMO.

A 2025 single-center retrospective study (*n* = 24) reported that 54.2% of patients with trauma receiving ECMO required blood transfusions, indicating a high incidence of bleeding. In the same cohort, the incidence of DVT was 12.5%, and DIC occurred in 8.3% of patients [28]. Additionally, 45.8% of patients experienced transfusion-related complications, and limb ischemia was observed in 8.3%. Among the four patients who did not receive anticoagulation, two still required blood transfusions; however, no other ECMO-related complications were reported.

Importantly, in the subset of patients with TBI (*n* = 7), no significant increase in intracranial hemorrhage was observed during ECMO support, providing further support for the feasibility of individualized anticoagulation strategies in this population [28].

Rates of oxygenator thrombosis were reported between 19% and 22.9%, with circuit thrombosis accounting for approximately 13.4% of cases [21]. Due to the unique hemodynamic characteristics of VA ECMO, the risk of thrombotic complications is higher than with VV ECMO [28]. Accordingly, optimal anticoagulation management, regular ECMO circuit inspections, and early identification of high-risk patients are essential to prevent thrombotic events.

### Individualized anticoagulation strategy

In patients with trauma, the concurrent risks of hemorrhage and thrombosis present substantial challenges to anticoagulation management during ECMO. Standardized protocols generally aim to maintain an ACT between 180 and 220 s or an aPTT of 40 to 80 s [2]. However, these fixed targets may not be appropriate for the complex and evolving clinical conditions observed in this population. As a result, individualized anticoagulation strategies that respond dynamically to changing risk profiles are necessary.

Recommendations for bleeding risk stratification, based on the ELSO and trauma-ECMO consensus, are summarized in Table 1, while comparative anticoagulation strategies are outlined in Table 2 [30–35].

**Table 1:** Bleeding risk stratification recommendations

Risk level	Clinical characteristics	Anticoagulation management recommendations
High risk	Active intracranial hemorrhage, within 24 h post-operative, coagulation factors < 30%	Use heparin-free strategy or delayed anticoagulation (> 48 h) whenever possible
Moderate risk	Hepatic dysfunction, mild brain injury, extensive soft tissue contusion	Low-dose heparin protocol with dynamic adjustment may be adopted
Low risk	No active bleeding, normal coagulation function	Standard-dose heparin protocol may be used

**Table 2:** Anticoagulation strategy comparison (specific dosage and monitoring parameters)

Type	Heparin dosage/target	Target population	Monitoring frequency
Heparin-free ECMO strategy	Use heparin-coated circuit without additional heparin; maintain flow rate 4–5 L/min	High bleeding risk, traumatic brain injury patients	Assess oxygenator pressure gradient + D-dimer every 8 h
Low-dose heparin protocol	ACT maintain 120–140 s or aPTT 35–50 s	Moderate bleeding risk patients	ACT every 6–8 h,
			aPTT once daily,
			D-dimer and platelet count once daily
Standard-dose heparin protocol	ACT 180–220 s or aPTT 40–80 s	Those without significant bleeding risk	Same as low-dose

Several adjunctive measures may support thrombosis prevention in patients with trauma undergoing ECMO. Real-time monitoring of circuit parameters, such as changes in oxygenator pressure gradients, circuit conductivity, or blood pump acoustics, can aid in the early detection of thrombus formation. Maintaining adequate perfusion is also essential; blood flow rates exceeding 4.5 L/min are recommended to reduce the formation of low-flow regions that predispose to thrombosis. In patients with a prior history of venous thromboembolism or rising levels of fibrin degradation products, routine ultrasound screening for DVT is advised. Additionally, circuit integrity should be preserved through daily inspection for air bubbles and scheduled replacement of monitoring connectors every three days.

Patients with trauma often present with overlapping high-risk factors such as hemorrhagic shock, visceral organ rupture, and craniocerebral injury [21, 36, 37]. Despite the elevated bleeding risk, a baseline level of anticoagulation remains necessary to prevent thrombus formation within the extracorporeal circuit. This clinical scenario requires a carefully managed balance between anticoagulation and hemostasis.

Currently, authoritative guidelines provide limited recommendations regarding optimal initiation timing and dosing strategies for “heparin-free” or “low-dose heparin” regimens in this population. The absence of standardized protocols contributes to marked variability in anticoagulation practices across centers. Further evidence-based guidance is needed to support clinical decision-making and to reduce complications related to both thrombosis and bleeding.

## ECMO prognosis and influencing factors

Although ECMO provides a critical life-sustaining intervention for patients with severe trauma, clinical outcomes remain highly variable. Reported survival rates under ECMO support range from 33.3% to 74.1% [11, 14, 21], reflecting the multifactorial nature of prognosis. Outcomes are determined not only by ECMO-related technical parameters but also by baseline patient characteristics, the timing of ECMO initiation, the selected ECMO modality (veno-venous vs. veno-arterial), and the quality of complication management.

### Survival rate differences and sources of heterogeneity

Large-scale data suggest that the overall survival rate among patients with trauma receiving ECMO support is approximately 46.1% [21]. Within this population, those with isolated respiratory failure experience significantly lower mortality (22.8%), whereas patients with circulatory failure or multiple organ dysfunction have substantially higher mortality rates, reaching up to 55.9% [21]. These findings, however, are primarily drawn from retrospective analyses and single-center studies, which are inherently limited by selection bias and incomplete data integration. High-quality evidence from large-scale prospective RCTs remains unavailable (Level B–C).

The wide variation in reported survival rates reflects substantial heterogeneity in study design, patient populations, and clinical settings. The primary contributors to this heterogeneity include the following factors [11, 14, 38, 39]:

Timing of ECMO initiation: Early initiation (< 48 h) is associated with improved outcomes, while delayed initiation (≥ 72 h) may reduce survival by nearly half.

Variation in ECMO indications: VV ECMO is primarily indicated for ARDS, whereas VA ECMO is often employed in cardiogenic or hemorrhagic shock—conditions associated with higher baseline risk.

Differences in trauma severity: Greater variability in ISS or NISS is observed across studies, with poorer outcomes in patients presenting with coexisting sepsis, coagulation dysfunction, or TBI.

Support environment variability: Prognosis may be influenced by whether ECMO is administered in high-volume centers and the degree of experience among ECMO teams.

A critical appraisal of this heterogeneity is essential to accurately evaluate the generalizability of clinical findings and to inform evidence-based application of ECMO in trauma care.

### Key clinical factors affecting prognosis

#### Anatomic and physiologic scoring systems

Although the ISS is widely used in trauma assessment, the NISS, which aggregates the scores of the three most severely injured anatomical regions, has demonstrated greater sensitivity in evaluating ECMO candidacy. Patients with trauma presenting with high NISS values often exhibit multi-system injuries and face a significantly elevated risk of mortality [14].

**Fig. 1:**
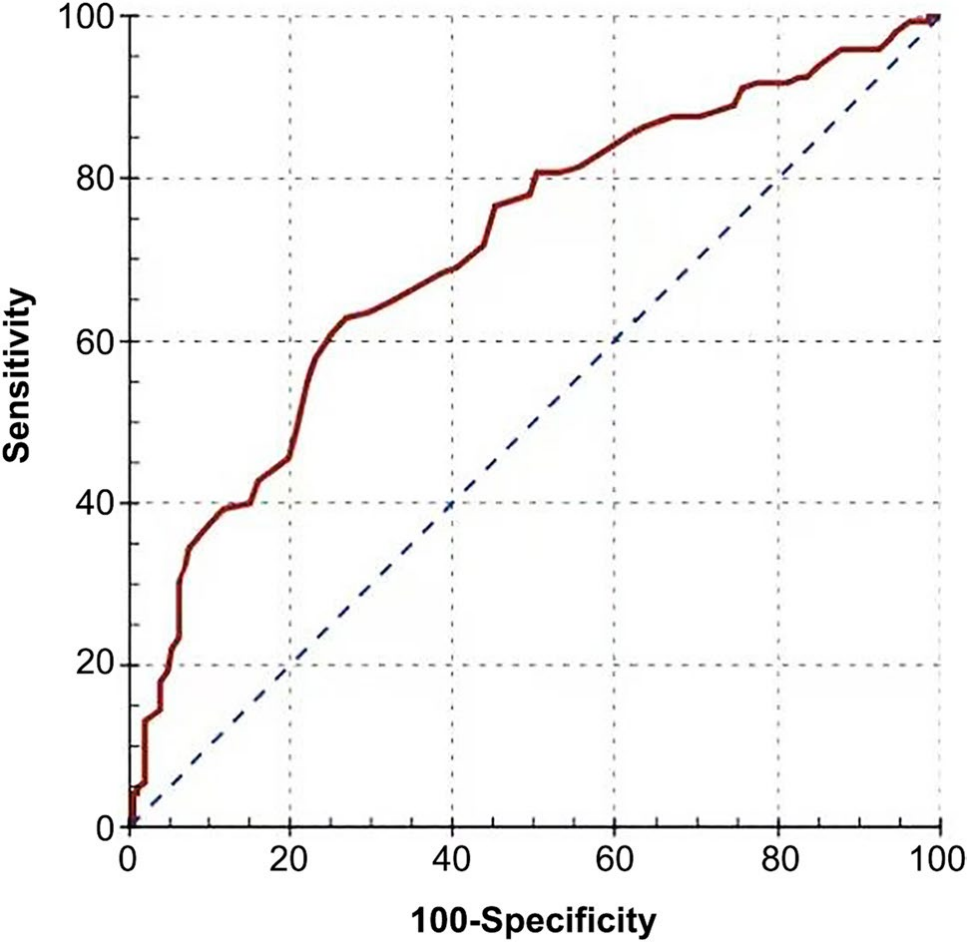
Prognostic performance of the SAPS III score in patients with trauma receiving ECMO support. A SAPS III score > 69 is associated with increased mortality risk. (AUC = 0.712, *p* < 0.001; sensitivity = 62.8, specificity = 73.1.)

The Simplified Acute Physiology Score III (SAPS III) provides a robust measure of early physiological status in critically ill patients. Each incremental increase in SAPS III is associated with a 4% rise in mortality risk (HR 1.04; 95% CI, 1.02–1.06; *p* < 0.001). A SAPS III score >69 has been identified as an optimal threshold for predicting mortality (area under the curve [AUC] = 0.712; *p* < 0.001) [39] (see Fig. 1).

Additional clinical variables have been independently associated with adverse outcomes in patients with trauma receiving ECMO. These include ECMO initiation due to hypothermia (HR 3.75; 95% CI, 1.64–8.56; *p* < 0.001), initiation during weekends (HR 1.60; 95% CI, 1.09–2.34; *p* = 0.016), and the occurrence of bleeding complications during ECMO support (HR 1.74; 95% CI, 1.26–2.41; *p* = 0.001). A SOFA score >12 is also strongly associated with increased risk of multi-organ dysfunction and may serve as a reference point when evaluating ECMO initiation [39].

#### Risk factors for patients with trauma receiving ECMO

**Fig. 2:**
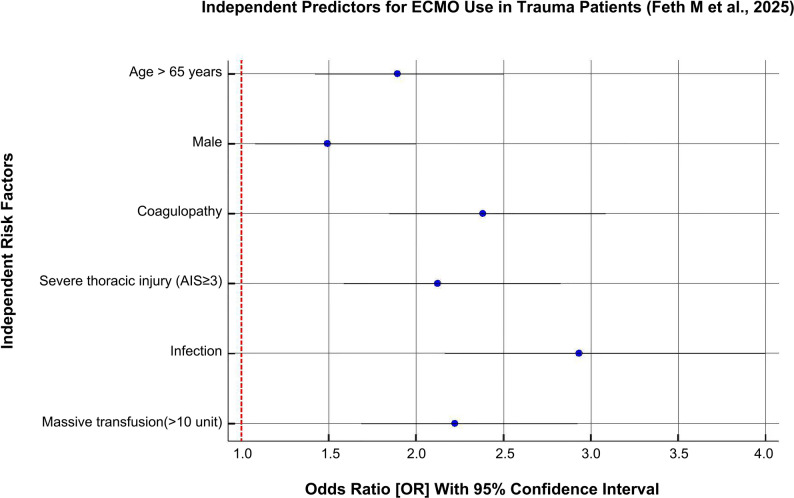
Independent predictors of poor prognosis in patients with trauma receiving ECMO support

A retrospective analysis by Feth et al. (2025), based on data from the TraumaRegister DGU^®^, identified several independent risk factors associated with increased mortality in patients with trauma undergoing ECMO support [21]. These include: age >65 years (OR 1.90), male sex (OR 1.49), severe chest trauma defined by an Abbreviated Injury Scale score ≥ 3 (OR 2.12), massive transfusion exceeding 10 units (OR 2.23), and the presence of coagulation dysfunction at baseline (OR 2.37). In addition, the development of complications during ECMO, specifically hemorrhage (HR 1.74) and infection (HR 1.82), was also associated with a significantly increased risk of mortality (see Fig. 2).

#### Importance of initiation timing

Multiple studies have demonstrated that early initiation of ECMO—typically defined as within 48 h of trauma—is associated with significantly improved outcomes compared to delayed initiation. A retrospective analysis reported a 30-day survival rate of 64% among patients with trauma who received ECMO within 48 h, in contrast to a survival rate of only 20% among those for whom initiation was delayed beyond 72 h [40].

These findings highlight the critical importance of early identification of high-risk patients and the timely initiation of ECMO support. Initiating ECMO before the onset of irreversible respiratory or circulatory failure may improve overall prognosis and reduce the risk of complications.

## Application challenges and future directions of ECMO in patients with severe trauma

While ECMO presents a promising therapeutic option for patients with severe trauma, its clinical application continues to face several critical challenges. These include procedural complexity, intensive resource requirements, and difficulties in perioperative management. Broader systemic limitations, such as the lack of standardized trauma-specific clinical guidelines, unresolved ethical considerations, and a predominance of low- to moderate-quality evidence, further hinder consistent implementation. Addressing these barriers is essential to advancing the safe and effective integration of ECMO into trauma care.

### Technical and management challenges: from “organ support” to “precise management”

#### Catheterization difficulty and conflict with trauma sites

Patients with major trauma present with multi-regional injuries involving the thorax, abdomen, pelvis, or soft tissues. Anatomical disruptions such as open wounds, rib fractures, chest wall instability, or subcutaneous emphysema may limit the feasibility of standard ECMO cannulation approaches, including femoral vein–jugular vein or femoral artery–jugular vein access routes [41]. In these settings, vascular access may require traversal through injured tissue, thereby increasing the risk of complications such as pseudoaneurysm formation, hematoma, vessel perforation, or inadvertent cardiac or pericardial injury.

Moreover, trauma-associated abdominal compartment syndrome can lead to elevated intra-abdominal pressure, impairing venous return and reducing ECMO circuit flow, with the potential for partial or complete circuit collapse.

To mitigate these risks, individualized cannulation strategies are recommended. Image-guided techniques, such as bedside ultrasonography, transesophageal echocardiography, or fluoroscopic/X-ray guidance, should be employed to improve procedural accuracy and reduce the incidence of access-related complications [42].

#### Insufficient standardization of infection complication management

Although infection control guidelines, such as those issued by the ELSO and various expert consensus statements, provide general recommendations on circuit maintenance and infection prevention, their applicability to patients with trauma has not been thoroughly validated. Several practices, including early microbial screening, probiotic administration, and protocols for the frequency of oxygenator replacement, remain largely based on single-center experiences. These approaches currently lack support from high-quality, multicenter clinical trials, thereby limiting their generalizability to trauma populations [37].

#### Uncertainty in initiation timing and risk of overtreatment under complex conditions

In the context of high trauma severity and multi-organ dysfunction, determining the optimal timing for ECMO initiation, as well as appropriate criteria for discontinuation, remains a major clinical challenge. Current decision-making is often extrapolated from models developed for acute respiratory distress syndrome (ARDS), which do not fully account for the pathophysiological heterogeneity characteristic of trauma populations [43].

The absence of standardized, trauma-specific algorithms contributes to variability in clinical practice. In certain cases, patients may rapidly deteriorate and develop MODS shortly after ECMO initiation, raising concerns regarding premature initiation or inaccurate indication assessment.

### Medical ethics and resource allocation challenges

While ECMO may provide survival benefits for patients with severe trauma, its use raises significant ethical and resource allocation concerns [44]. Key challenges include establishing appropriate patient selection criteria, ensuring informed consent under emergency conditions, and promoting equitable access to ECMO in resource-limited settings [45].

In trauma care, ethical decision-making must weigh the potential benefits of ECMO, such as increased survival with early initiation, against its inherent risks, including procedural complications and long-term neurological impairment. This is particularly relevant for patients with TBI or multiple organ dysfunction. A 2025 expert consensus highlights the importance of involving multidisciplinary ethics committees (MECs) in ECMO-related decisions to uphold transparency and patient-centered care principles [46].

Resource allocation remains a core issue, especially in low-resource environments. The ELSO recommends prioritization based on clinical scoring systems such as the SOFA and the Respiratory Extracorporeal Membrane Oxygenation Survival Prediction (RESP) score, with preference given to patients with reversible pathology and preserved baseline function [27]. In many trauma centers, limited ECMO equipment and staffing contribute to allocation disparities. For instance, ECMO utilization rates in resource-constrained hospitals are approximately 60% of those in high-volume centers, contributing to a reported 15% difference in survival outcomes [44]. The implementation of regional ECMO networks and remote consultation platforms has been proposed to optimize resource distribution.

Ethical considerations are especially complex in the context of extracorporeal cardiopulmonary resuscitation (ECPR) for TCA. A 2025 review emphasizes the need for careful evaluation of prognostic uncertainty, such as the potential for neurological recovery below 50%, and advocates for dynamic, ongoing communication with family members during consent processes [46].

Ethical frameworks for trauma ECPR should include: (1) defined eligibility criteria (e.g., low-flow time < 60 min); (2) consideration of post-ECMO quality-of-life implications (e.g., long-term ECMO dependence); and (3) prioritization of younger patients with fewer comorbidities to promote fairness [46]. For patients with TBI and TCA, careful balancing of anticoagulation risks and benefits is critical, and MECs can provide structured guidance in such high-risk scenarios [21].

The development of ECPR-specific ethical guidelines is necessary to ensure that patient values, clinical appropriateness, and long-term resource sustainability are integrated into ECMO decision-making. These considerations not only shape bedside practice but also inform the design of future multicenter clinical trials.

### Insufficient clinical evidence: lack of high-quality prospective study support

Current evidence regarding the use of ECMO in patients with severe trauma remains limited, with most studies based on retrospective cohort analyses, single-center experiences, or case series. The absence of RCTs significantly restricts the strength of clinical recommendations, which are largely informed by expert consensus or observational data rather than high-level (Grade A) evidence [23, 28].

Several critical aspects of ECMO implementation in trauma populations, such as the optimal timing of initiation, risk-based stratification of anticoagulation strategies, evaluation of infection control interventions, and long-term neurological outcome assessment, lack standardized quantitative indicators and validated assessment frameworks. This persistent deficiency in high-quality prospective data impedes the development of evidence-based protocols and limits both the reproducibility and generalizability of clinical decision-making in trauma-related ECMO support.

### Future research directions and outlook

#### Development of trauma-specific ECMO decision-making models

Future research should prioritize the development of decision-support tools tailored to trauma populations. Integrating established clinical indicators such as the SOFA score, NISS, and serum lactate levels with coagulation profiles, inflammatory markers, and ECMO-related parameters may facilitate the development of trauma-specific scoring systems. The incorporation of machine learning techniques, such as random forest algorithms and deep neural networks (e.g., eCMoML, ECMO PAL), offers a promising approach to enable real-time, individualized assessment of ECMO eligibility and prognosis [47, 48]. These models can support balanced risk–benefit evaluations and improve clinical decision-making under complex and time-sensitive conditions.

#### Advancement of multi-center prospective studies

To address the persistent limitations in evidence quality, there is an urgent need for well-designed, multi-center prospective studies, including RCTs and large-scale cohort investigations. Future research should focus on several priority areas critical to optimizing ECMO application in trauma care. These include the comparative evaluation of ECMO modalities, particularly veno-venous versus veno-arterial configurations; the refinement of anticoagulation strategies, such as the use of standard-dose versus heparin-free or low-dose regimens; and the systematic development of protocols for the prevention and management of complications, especially infection and thrombosis [49].

#### Integration of AI-assisted diagnosis and intelligent monitoring technologies

The application of artificial intelligence (AI) in the management of critically ill patients is undergoing rapid development, particularly in intensive care and extracorporeal support settings [47, 50]. In the context of ECMO therapy for trauma, AI-driven algorithms hold potential to enable real-time assessment of perfusion adequacy, oxygenation levels, and individualized bleeding and thrombotic risk stratification. Through continuous data integration and predictive modeling, such technologies may significantly enhance the precision of clinical decision-making, facilitate earlier intervention, and improve the efficiency of patient monitoring and resource allocation.

#### Development of trauma-specific ECMO clinical pathways and guidelines

To promote consistency and evidence-based practice in the use of ECMO for patients with trauma, there is a clear need for the establishment of dedicated clinical pathways and standardized guidelines. It is recommended that international expert bodies, such as the ELSO, lead these efforts by consolidating current evidence and expert consensus. The resulting guidelines should comprehensively address key areas including patient selection criteria, timing and modality of ECMO initiation, anticoagulation strategies, criteria for weaning and termination, and ethical considerations. Such frameworks would support harmonization of ECMO practice across institutions and regions [50–52].

## ECMO in resource-constrained environments

In healthcare institutions with limited access to ECMO, allocation decisions present complex ethical challenges that require a framework grounded in equity, transparency, and clinical utility. Effective allocation must balance multiple competing considerations while avoiding arbitrary or discriminatory practices.

Central to this decision-making process is the assessment of clinical prognosis, particularly the likelihood of survival and meaningful recovery. Objective, validated tools such as the RESP score and the SOFA score are recommended to identify patients with the highest probability of deriving significant benefit from ECMO support.

Urgency must also be considered. Patients at immediate risk of death who have potentially reversible conditions should be prioritized, as ECMO may provide a critical window for recovery. In parallel, resource efficiency is a practical concern. Patients expected to require shorter durations of ECMO support may be favored in order to maximize the number of lives saved over time.

To minimize bias and ensure consistency, such ethically sensitive decisions should not rest solely with individual clinicians. Instead, an independent, multidisciplinary triage committee, comprising critical care specialists, trauma surgeons, ethicists, and where feasible, community representatives, should be established to apply predetermined criteria, offer diverse perspectives, and uphold procedural fairness. This approach fosters legitimacy and mitigates variability in decision-making.

Transparency in both process and criteria is essential. Clear communication with healthcare teams, patients, and the public promotes trust and understanding, even when decisions are difficult. Regular auditing of outcomes, along with a willingness to revise protocols in response to emerging evidence or contextual changes, is essential to maintaining the fairness and effectiveness of ECMO allocation strategies.

Ultimately, a structured, ethically informed framework allows institutions to navigate resource limitations while prioritizing clinical outcomes, maximizing benefit, and upholding fundamental principles of justice and accountability.

## Conclusion

ECMO provides a critical supportive modality in the management of severe trauma, including trauma-associated ARDS, circulatory failure, and cardiac arrest, particularly when conventional therapies fail. Available evidence indicates that VV ECMO is associated with higher survival rates than VA ECMO, and early initiation may improve outcomes. However, current data are predominantly derived from observational studies, highlighting the need for further validation.

To enhance the safe and effective application of ECMO in trauma care, future efforts should focus on developing trauma-specific decision tools, conducting high-quality multicenter prospective studies, and establishing standardized protocols for anticoagulation and infection control. These steps are essential to support individualized treatment and improve clinical outcomes in this complex patient population.

## Data Availability

All data generated or analysed during this study are included in this article. Further enquiries can be directed to the corresponding author.

## Abbreviations

ECMO: Extracorporeal membrane oxygenation
VV ECMO: Veno-venous extracorporeal membrane oxygenation
VA ECMO: Veno-arterial extracorporeal membrane oxygenation
ARDS: Acute respiratory distress syndrome
VILI: Ventilator-induced lung injury
TBI: Traumatic brain injury
ECPR: Extracorporeal cardiopulmonary resuscitation
TCA: Traumatic cardiac arrest
SOFA score: Sequential Organ Failure Assessment score
MODS: Multiple Organ Dysfunction Syndrome
VAP: Ventilator-associated pneumonia
ICU: Intensive care unit
CRP: C-reactive protein
PCT: Procalcitonin
ACT: Activated clotting time
aPTT: Activated partial thromboplastin time
D-dimer: Fibrin degradation product D-dimer
ELSO: Extracorporeal Life Support Organization
ISS: Injury Severity Score
NISS: New Injury Severity Score
TNF-α: Tumor necrosis factor alpha
IL-6: Interleukin-6
SIRS: Systemic Inflammatory Response Syndrome
SAPS III: Simplified Acute Physiology Score III
ACS: Abdominal Compartment Syndrome
